# Foreign Letter

**Published:** 1890-05

**Authors:** Floyd S. Crego

**Affiliations:** Berlin


### FOREIGN LETTER.

BERLIN, February 25, 1890.

*Editors Buffalo Medical and Surgical Journal:*

ONE of the greatest pleasures I have had since my stay in Berlin is the attendance on the regular weekly meetings of the Berlin Medical Society. Several papers are presented every evening, and one has an opportunity to hear such men as Leyden, Virchow, Frankel, Mendel, Martin, Olshausen, Litten, Senator, and many others quite as eminent. It is needless for me to state that to hear such men every member tries to be present, and usually by half-past seven, the hour for meeting, there are three to four hundred out of the seven hundred members present. Prof. Virchow is the president. The subject discussed for two meetings was Extra-uterine Pregnancy, and it seemed to me from reading recent literature, that much that was new and of the greatest interest, was brought forth by the paper and from the discussion. Prof. Olshausen read the paper. He reported cases of twin extra-uterine pregnancy, and also called attention to the fact that it is apt to occur a second time in the same patient. The diagnosis is easy at the first month. The menstruation most always ceases at first and often recurs. In the second stage there is no difficulty about diagnosis and can be made with certainty (?) Tubular extra-uterine pregnancy is by far the most common, as it occurs ten times as often as any other. Many cases of supposed abdominal pregnancy were undoubtedly tubular. What was formerly supposed to be abdominal pregnancy is now known to be tubular; the placenta being attached to the inner part of the tube, and the fetus grows into the cavity after rupture of the tube. Primary abdominal pregnancy may, however, exist. The treatment of these conditions is the main consideration, and I pass on to this. If rupture of the tube has not taken place, there is no safer operation than laparatomy, and this should be resorted to as early as first or second month. If rupture has occurred, the state of the patient determines whether to operate or not. If a few days have already elapsed since rupture, and hematocele is formed, do not operate. In one case he had operated twice; at the first operation the placenta was not detached, but in eleven days the second operation was undertaken and the placenta was easily removed. Another case, diagnosis was made in fourth month, but the patient would not allow operation until two weeks before the full time. Laparatomy was performed then, the child born alive, but died soon afterwards. Placenta was not removed. Patient did well until thirty-four days after this, when she suddenly became comatose, remained in coma fourteen days and died. In another case, childs position changed so that it was suspected that a rupture had taken place. It was found to be true upon operation. At firsr when cavity was opened, nothing could be seen but intestines, on searching the child was found buried in the intestines and was born alive. The sac could not be seen. The child and mother lived. In a few years mother returned with a second extra-uterine pregnancy, on the other side of the body. The sac should be removed if possible; if not, then the placenta, providing there is not too much bleeding. If there be danger from hemorrhage by removing the sac, it is better to leave it in the cavity. In general the operation is easy. It should be performed for the benefit of the life of the mother, the life of the child being secondary; the child rarely lives if born alive. In former years when the child was viable and operation performed, out of twenty-two cases nineteen died. The old idea of waiting ten weeks until after the death of the child was erroneous, and killing child by puncture or electricity was quite as false. In cases of rupture, if the bleeding continues, operate at once; if hematocelé has formed, wait. If the child is viable, operate.

### DISCUSSION.

Prof. MARTIN said: I will say a word concerning this most interesting condition, chiefly because the reader of the paper has in two places quoted me in a somewhat different way than I desire to go on record. He quoted me as saying some years ago that the diagnosis of extra-uterine pregnancy was quite difficult. Since that time, I have had opportunity to see many cases of extra-uterine pregnancy. I have operated twenty-two times, and have seen many cases that did not come to operation. I was of the opinion once that it was somewhat easier to make the diagnosis in such cases than I had at first maintained, but I am sorry to say that I turn more and more to the opinion first expressed, that this condition is not easy to diagnose, especially in the early months. In later months, when we can feel the child, is it easy to diagnose? Prof. Olshausen has said the menstruation is a guide to the diagnosis, although he said it was somewhat unreliable, but I am of the opinion there is not much reliance to be placed on this symptom. My first case was a woman who had never once missed the regular menstruation. In this case I performed laparatomy, and removed a child. At this time she had never spent a day in bed. The tumor was somewhat large and hindered her in working at her trade, that of sewing with a machine; on this account she desired the tumor removed. He reported two more cases to illustrate this point; both of these cases came under his care after rupture of the tubes with severe hemorrhage. Both were operated on. Many times we can make the diagnosis by bimanual palpation, but we can mistake this condition for tubular and ovarian troubles, as in these diseases the menstruation is often irregular. I must say the diagnosis of extra-uterine pregnancy is not easy, and especially not in the early months of pregnancy.

With very especial pleasure I have listened to the therapy of extra-uterine pregnancy, as presented by the reader. You know that especially Winckel has returned to the theory expounded by Friedriech, that the fetus should be killed by injection, and then wait for its elimination in one way or another. I do not consider this good advice, but take the same stand as Professor Olshausen, that the sac should be removed, and, also, that the *whole* sac should be removed. In 1881, when I presented this view to the International Congress in London, I found considerable opposition. At that time it was thought impossible to remove the sac without great difficulty, especially in abdominal pregnancy, but we know now that abdominal pregnancy is a very uncommon thing, and that in tubular pregnancy we have the chance to remove the sac. I also then, and in 1884 in Copenhagen, pleaded that the placenta under all circumstances should be removed, and gave the advice to ligate. I admit that there are cases in which this is very difficult. I maintained then that the sac could not always be removed, but that instead of sewing it on to abdominal walls, to close the abdominal opening and drain through the vagina. I agree with the reader that operation as early as possible is the only way to deal with this condition and relieve the mother of months of suffering.

Discussion was further carried on by Privat-docent Landau, who had had nineteen cases with but two deaths. He agreed with the reader that the diagnosis was not difficult, but yet he had once made a mistake in diagnosis.

Dr. Czempin took decided grounds against the reader, as he maintained it was not easy to diagnosticate this condition early. Many cases of mistaken diagnosis were reported by competent men. He considered that a very good point in diagnosis was the growing of the uterus along with extra-uterine pregnancy, as we know it does grow to about the size of a four months pregnant uterus, then could be felt the two tumors side by side. Professor Virchow presented several specimens and called attention to the fact that in old cases the child itself never calcifies, but that the sac is filled with lime salts and gradually hardens and builds a wall about the child. My oldest case of twenty-six years standing proved these assertions. It was possible after this time to recognize the form of the child and also under the miscroscope to distinguish the histological character of the same.

The nervous clinics continue to be quite as interesting, and rare cases are presented nearly every day along with typical cases of nervous disorders frequently seen. A case of Basedow's disease in man was recently presented. It was caused by fright two years before. The neck became enlarged, heart irregular, and eyes protruded, sleepless at night, sweats constantly. Was improving under milk cure and rest at the bath resort, Cordova. Several cases of what Westphal termed abortive insanity were presented. Case I.—Patient fears she will injure herself or her friends. When she crosses a bridge can hardly resist the temptation to jump into the water; Case II.—Young man who cannot work because everything he touches or comes in contact with he must thoroughly clean; when once cleaned he repeats this over and over again. In passing a show window, if he takes one glance at it he must return to it. He dusts his room over and over again, etc.; Case III.—A cashier who had to count his money over and over again. Many persons in this condition utter obscene words against their will if they have chanced to hear them from some one else just previously. Perhaps they will struggle to suppress it for a day or so, but finally will repeat it. Heart disease was noticed in the cases. Westphal first described them, and said they rarely developed insanity, and were not fit subjects for an asylum as they rarely injure themselves or others, and usually run a long course and recover. The beginning is sudden and the termination just as sudden. Such cases should be treated by travel, cold water cure, sea baths and nerve tonics. Professor Mendel showed a child eleven years of age, with multiple sclerosis cerebri; head is in constant motion, pupils widely dilated, partial facial paralysis in the right side. On account of progressive paralysis she is unable to stand; mind affected; patellar reflexes exaggerated. He reported this case and showed that only within the last few years was this condition noted in children. The prognosis was unfavorable, as the time allotted for it to run its course to a fatal termination was three years. It was well to use electricity and iodides; using constant current to the head; exercising great care to use a weak current with the exact strength measured by a good galvanometer. A disease described by Charcot very recently, called "maladie de tics," proved very instructive from a therapeutic standpoint; an unfavorable prognosis was formerly given, but Mendel succeeded in relieving three cases almost immediately by the use of tinct. gelsemium in small doses:

CASE I.—Miss C., aged 23, jerks the head constantly, knits her brows, muscles of the face twitch, now and then the patient utters a loud noise, or speaks a word as if it were an echo, or does repeat the last word of any sentence heard.

CASE II.—Miss J., aged 14, has continual muscular tremor all over the body almost like chorea of worst form. Makes a half crying, half sighing noise. At times utters meaningless words or sounds. At night these movements cease.

CASE III.—Miss B., aged 12, almost similar case. Face, red; pulse, very rapid.

All these cases were of long standing. The exact character of these cases was not understood. Hypnotism, arsenic, cold water cure did these patients no good, as they had all been under treatment, but the use of tinct. gelsemium in three drop doses three times a day relieved the patient at once. There were two wonderful cases of hysteria recently. A man came to the policlinic on crutches; had not walked for a year; sight was imperfect; had unsound sleep, etc.; had lost in weight. Several off-hand diagnoses were made by the assistants, most leaning to the theory of alcoholic paralysis. Taking the crutches away, patient in five seconds commenced to tremble violently from head to foot and finally fell down. This proved to be hysteria. Professor Mendel said he proposed to treat this patient by suggestion without hypnotism. Patient was treated with large magnet placed across the legs for fifteen minutes; was not allowed to talk or move during this time, and at the end of treatment could walk a step or two. Has since continued to improve. Second case—a woman presenting the following symptoms: Had not eaten for eight days; the eyes were wide open and conjunctiva red; claimed she could see nothing; had not shut her eyes for eight days or so; as far as known had not moved the eyeballs. If this condition had depended on an organic lesion it would have been so extensive that the patient could not have walked. She might have had hemianopsia (or half vision), but to have complete loss of vision, with paralysis of six and eight nerves, was impossible without graver symptoms, paralysis of extremities, etc. This case was also treated with same method, suggestion without hypnotism. A magnet was placed on side of head, held firmly against the temporal region. Soon the patient felt the current, she said, then she saw a dim light, then could see, then closed the eyes, then could move the eyeballs. She left the clinic fully healed and returned the next day to show that she was still well. Professor Mendel applies hypnotism to the treatment of nervous conditions, but thinks it is much like the use of morphine; of some value in many cases, but to be used with great caution. He described many cases of hypnotism mania cases following the constant application, in which this only would help. In my next letter I will describe some of the spinal diseases presented by Dr. Oppenheim and Professor Mendel, and give a differential diagnosis of the same; and also, some points in alcoholic neuritis, which is exceedingly common here.

FLOYD S. CREGO.

